# PROTEOMIC BRAIN AGE GAP, DEMENTIA RISK, AND BRAIN VOLUME MEASUREMENTS

**DOI:** 10.1093/geroni/igae098.3470

**Published:** 2024-12-31

**Authors:** Minghao Kou, Hao Ma, Xuan Wang, Lu Qi

**Affiliations:** Tulane University, New Orleans, Louisiana, United States; Tulane University, New Orleans, Louisiana, United States; Tulane University, New Orleans, Louisiana, United States; Tulane University, New Orleans, Louisiana, United States

## Abstract

Aging is the most profound risk factor for dementia, yet the molecular basis for brain aging and dementia remains largely unknown. We utilized levels of proteins primarily originating from the brain to measure brain-specific aging signature (age gap) and assessed its associations with all-cause dementia, Alzheimer’s disease (AD), and vascular dementia. We analyzed Olink Explore 1536 proteomics data from 45,113 UK Biobank participants, followed until December 31, 2022. Machine learning models estimated the brain age gap based on 53 brain-enriched proteins, demonstrating strong correlation with chronological age (Spearman r = 0.84). Per-unit increment in the brain age gap z-score was associated with significantly elevated risks of all-cause dementia (hazard ratio [95% confidence interval], 1.82 [1.69-1.96]), AD (2.12 [1.88-2.39]), and vascular dementia (1.91 [1.58-2.31]), respectively. Notably, 2.1% of the study population exhibited extreme brain aging defined as brain age gap z-score <-2 or >2, correlating with over 4-fold increased risks of all-cause dementia and vascular dementia (4.39 [2.94–6.55] and 4.16 [1.27–13.64], respectively), and up to 6-fold increased risk of AD (HR 6.15 [3.35–11.28]). These findings were corroborated by Magnetic resonance imaging (MRI) assessments showing that a higher brain age gap aligns with cortex atrophy and increased white matter hyperintensity. Conclusively, the proteomic brain age gap serves as a meaningful indicator of brain structural degeneration and dementia risk, independent of chronological age.

